# OpenEvidence

**DOI:** 10.5195/jmla.2026.2247

**Published:** 2026-01-01

**Authors:** Suja Philip, Reghu Kurian

**Affiliations:** 1 suphilip2001@gmail.com, University of the West Indies, School of Clinical Medicine and Research, Nassau, Bahamas; 2 reghuphilip@hotmail.com, Princess Margaret Hospital, Nassau, Bahamas

## Abstract

**OpenEvidence**. AI based Medical Information platform. Released 2023. OpenEvidence Inc. Cambridge. Massachusetts. https://www.openevidence.com/; Founder& CEO: DR. Daniel Nadler. Free of cost for Healthcare Professionals. Registration is required to use Open Evidence.

OpenEvidence, an artificial intelligence (AI) assisted medical platform, was founded by Dr. Daniel Nadler. OpenEvidence was developed in collaboration with a team of physicians and computer scientists with a mission to offer reliable, unbiased and validated medical information to healthcare professionals at no cost [1]. OpenEvidence (OE) is available through its website (http://www.openevidence.com/ and features partnerships with prominent journals such as NEJM, JAMA and Lancet to ensure content of the highest quality. Since its launch in 2023, in collaboration with the Mayo Clinic Platform, it remains an essential and evident resource in the medical field. OE leverages natural language processing (NPL) to streamline research efforts, and deliver comprehensive evidence-based insights into diagnosis, treatment options, and overall patient care [1].

Artificial Intelligence is revolutionizing the healthcare industry, enhancing patient care, clinical decision-making, and professional development. There is increasing demand for trustworthy and easily accessible medical information among healthcare professionals. Open Evidence (OE) addresses this demand by systematically organizing global medical knowledge into an accessible and clinically useful format [1].

The Open Evidence browser-based search engine requires users to register to login with their professional ID credentials. However, currently, it is also available to medical students in the United States, who can access OE by using their medical school credentials [3].

## FEATURES

OpenEvidence serves as a fast and reliable tool for answering clinical questions, such as inquiries regarding treatment options, drug dosing, drug side effects, drug interactions, labs to consider, alternative treatments, and updated guidelines. It can also handle more complex tasks, such as preparing for mock exams, conducting research on a specific topic, finding evidence, and developing appropriate treatment plans. A new feature of OE includes email notification, alerting users to updates on previously asked questions, and ensuring continuous access to the latest information. Additionally OE is available in multiple languages, further expanding its accessibility.

Unlike other medical websites and databases that offer pre-prepared information, OE allows users to directly request evidence without needing to spend hours sifting through articles. OE is an excellent resource for physicians at the point of care; it assists physicians to quickly obtain answers from the peer-reviewed materials, utilizing current research that is reliable for medical practice. OE has implemented specific strategies to incorporate evidence-based and peer-reviewed materials from reputable journals, such as the Lancet, JAMA, and the New England Journal of Medicine.

Open Evidence complies with the HIPAA [3]. Verified NPI users, identified as HIPAA-covered providers, can earn AMA PRA Category 1 credit. CME Credit is awarded after reviewing your previous openevidence question and completing a brief learning assessment. CME Credit is accessible through their email, contact@openevidence.com [2].

OE is available through web browsers and as a mobile application on iOS and Android platforms, offering healthcare professionals a powerful resource for enhancing clinical decision-making and improving patient care outcomes [1]. OE is a free, accessible medical resource provided by the Mayo Clinic Platform that encompasses key medical journals, medical books, and authoritative sources [4]. Available exclusively to healthcare professionals, it offers 24/7 access with accuracy verification managed by OE developers. Medical students and clinicians can use OE to find answers quickly and perform differential diagnoses. The platform's ease of access and zero cost make it an invaluable tool for medical students, faculty members, and healthcare professionals. The OE's ability to handle direct questions and provide quick concise answers will assist medical students to use this tool as a personal tutor or study buddy. Medical librarians may recommend OE as the most suitable AI tool for healthcare professionals and faculty members, both for clinical practice and as a teaching assistant

OE is based on a large language model (LLM). Effective use of LLM-based AI tools requires precise queries and awareness of limitations, including “making things up” or hallucination. OE overcomes these limitations by using a retrieval-augmented generation-based Large Language Model that references established medical sources [6]. In addition, OE provides citation links, which allow users to verify information and reduce the risk of inaccurate information.

In this review, a few evidence-based questions were used to compare OE and ChatGPT. The findings indicate that both resources provide similar information; however, OE offers accurate, faster and concise responses. Given that healthcare relies not only on medical knowledge but also on trust, accuracy, authority, and currency, OE proves to be the superior option compared to ChatGPT.

**Table 1:** ChatGPT vs OpenEvidence

Features	Chat GPT	Open-Evidence
Access	General Public & Subscription Option	Healthcare Professionals & US Medical Students
Adaptation	General Medical Knowledge	Clinical Decision Making & Evidence based
Cost	Free & Paid subscription	Free for US Healthcare Professionals.
Citation	Not Current	Current
Focus	Conversational	Reliable Medical Information.
Quality of information	Detailed, Potential for error and hallucination.	Concise, Accurate, Timely & peer reviewed articles.
Purpose	General AI tool	Specialized Medical AI tool
Training Data	Broad & General Data	Reliable Medical

## LIMITATION

Like any AI-driven platform, OE has certain limitations. The accuracy and detail of responses depend on the clarity and specificity of user input. The OE website may experience occasional lag, but this issue does not affect the mobile application. Additionally, OE information is not peer-reviewed. Proper use requires human intervention, medical expertise, and specialized knowledge.

The company has also emphasized in the terms of use that “OpenEvidence does not offer medical advice, diagnosis, or treatment. Users must ensure that their questions do not contain protected health information (PHI) or any privacy-violating details” [1].

## CONCLUSION

OpenEvidence summarizes medical knowledge for physicians and healthcare workers. Despite the challenges that remain, including issues surrounding data privacy and ethical considerations, the potential benefits of OE indicate that AI will play a pivotal role in the future of healthcare. According to OE, approximately 25,000 US doctors have been signing up monthly since the platform's launch in 2023. This adoption indicates a demand for accessible, evidence-based tools in medical practice [7]. OE's global accessibility and multilingual capabilities have ensured its widespread usability. As with any medical resource, human expertise and clinical judgment are essential for the proper use of OE. This AI-integrated medical search engine is highly beneficial because of its rapid access to authoritative medical knowledge, ability to provide targeted answers, free availability, and round-the-clock accessibility. Combined with human medical expertise, OE has the potential to enhance healthcare.

OE is available through web browsers and mobile applications for iOS and Android platform [5], offering healthcare professionals a powerful resource for enhancing their clinical decision making and improving patient care outcomes. Medical librarians can confidently recommend this authoritative, freely accessible resource to medical students and healthcare professionals.
